# The Medical Bulletin Visiting List for 1891

**Published:** 1891-01

**Authors:** 


THE MEDICAL BULLETIN VISITING LIST FOR 1891. Philadelphia. F. A. Davis Publisher.

Looking over this little annual, we find that it is complete in every respect. The cut page of the visiting list is an excellent feature, and will be a great convenience to the physician, saving his re-writing the names of his patients every day of the month. The formulae of Hypodermatic medication will be found very useful indeed. We think that the publishers have very thoroughly met all indications, and that the book recommends itself to the profession.

